# ViralZone 2024 provides higher-resolution images and advanced virus-specific resources

**DOI:** 10.1093/nar/gkad946

**Published:** 2023-10-28

**Authors:** Edouard De Castro, Chantal Hulo, Patrick Masson, Andrea Auchincloss, Alan Bridge, Philippe Le Mercier

**Affiliations:** Swiss-Prot group, SIB Swiss Institute of Bioinformatics, CMU, 1 Michel Servet, 1211 Geneva 4, Switzerland; Swiss-Prot group, SIB Swiss Institute of Bioinformatics, CMU, 1 Michel Servet, 1211 Geneva 4, Switzerland; Swiss-Prot group, SIB Swiss Institute of Bioinformatics, CMU, 1 Michel Servet, 1211 Geneva 4, Switzerland; Swiss-Prot group, SIB Swiss Institute of Bioinformatics, CMU, 1 Michel Servet, 1211 Geneva 4, Switzerland; Swiss-Prot group, SIB Swiss Institute of Bioinformatics, CMU, 1 Michel Servet, 1211 Geneva 4, Switzerland; Swiss-Prot group, SIB Swiss Institute of Bioinformatics, CMU, 1 Michel Servet, 1211 Geneva 4, Switzerland

## Abstract

ViralZone (http://viralzone.expasy.org) is a knowledge repository for viruses that links biological knowledge and databases. It contains data on virion structure, genome, proteome, replication cycle and host-virus interactions. The new update provides better access to the data through contextual popups and higher resolution images in Scalable Vector Graphics (SVG) format. These images are designed to be dynamic and interactive with human viruses to give users better access to the data. In addition, a new coronavirus-specific resource provides regularly updated data on variants and molecular biology of SARS-CoV-2. Other virus-specific resources have been added to the database, particularly for HIV, herpesviruses and poxviruses.

## Graphical Abstract

**Graphical Abstract:**
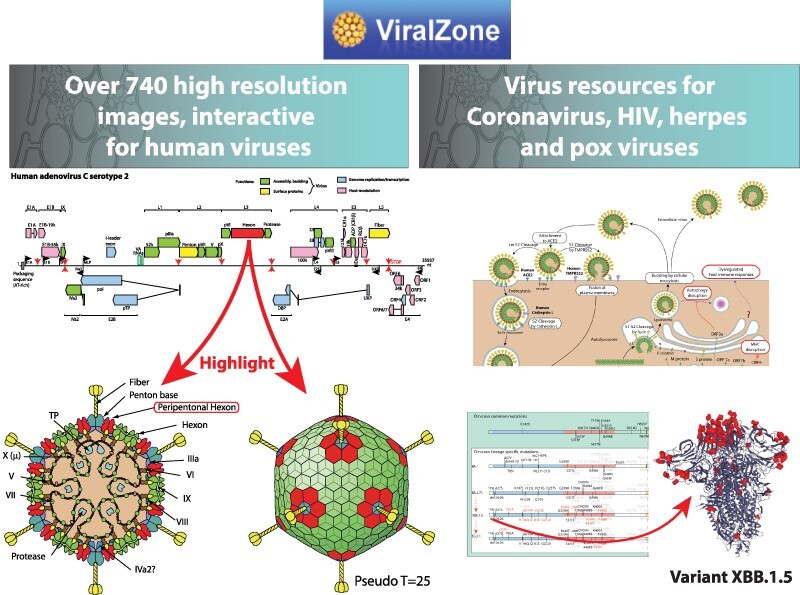

## Introduction

ViralZone is an online resource and database that provides information about viruses, particularly their molecular biology, taxonomy and the diseases they cause. It is a valuable tool for researchers, students and anyone interested in learning more about viruses. ViralZone provides a comprehensive taxonomy of viruses, classifying them into different families, genera and species according to the International Committee on Taxonomy of Viruses (ICTV) (1). The database contains 879 virus description pages with detailed descriptions of viruses from different virus families or genera, including their genome structure, replication cycle, host range and associated diseases. It also contains information on the proteins produced by viruses, their functions and their role in the viral life cycle; all are linked to UniProt entries (2). In addition, there are 352 pages describing viral molecular processes: from transcription, replication to icosahedral viral structure and host-virus interactions. All data come from publications, textbooks and feedback from experts to digitise the global knowledge of viruses. The ViralZone pages provide contextual links to many resources, the most important being: UniProt (2), National Center for Biotechnology Information (NCBI) (3), Virus Pathogen Resource (ViPR) (4), ICTV (1), Gene Ontology (GO) (5), RCSB Protein Data Bank (RCSB PDB) (6), Chemical Entities of Biological Interest (ChEBI) (7) and ChEMBL (8).

Overall, ViralZone provides educational materials and resources to help users learn more about virology and related topics. The database is maintained and regularly updated by the Swiss Institute of Bioinformatics (SIB) and is freely available to the public. The database has been enhanced by improving graphic resolution and making browsing more user-friendly. Special virus pages have been developed for important human pathogenic viruses, describing the molecular biology of a particular virus in great detail.

## Improvement in graphics and dynamic contents

**Figure 1.**
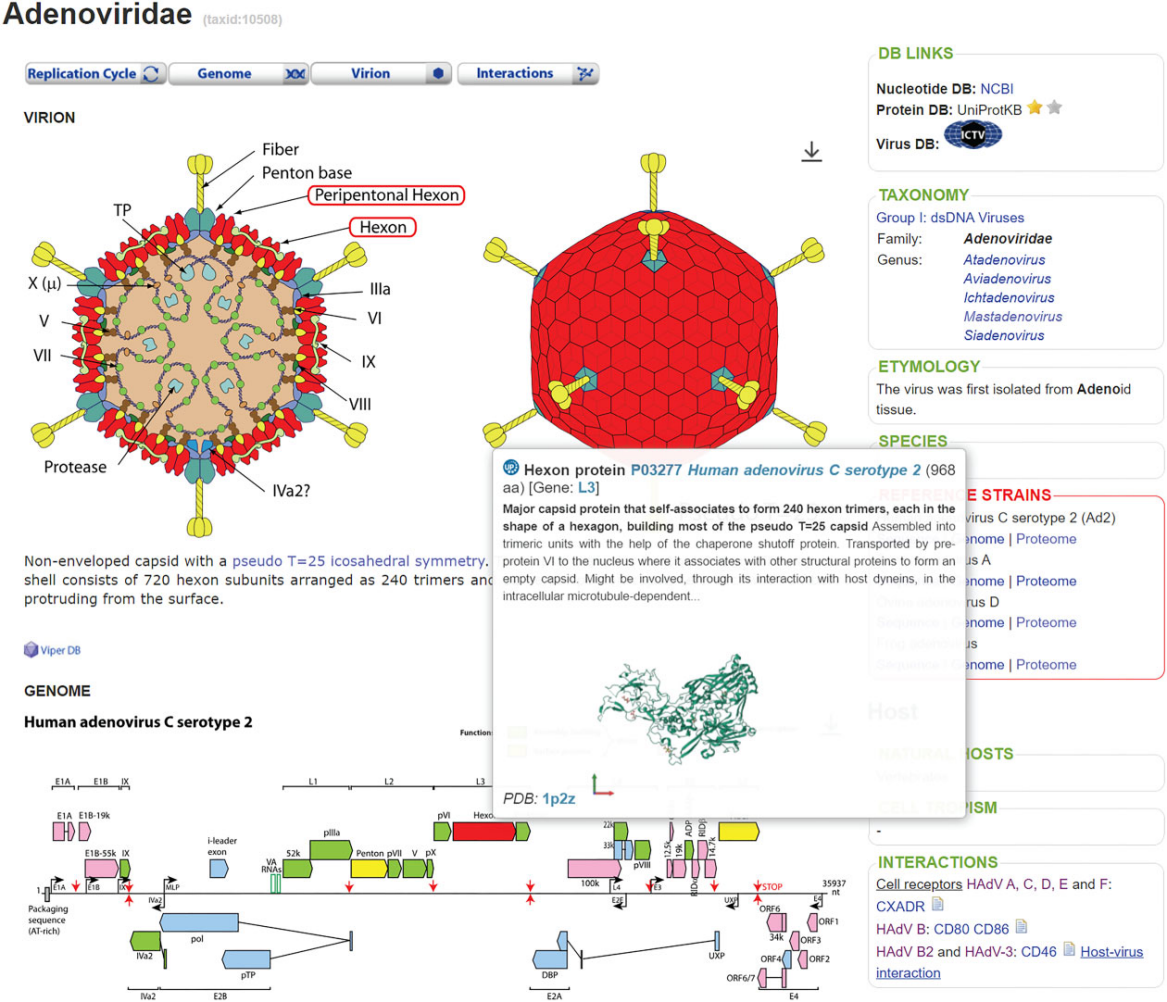
Images of the adenovirus virus and genome map. Hovering over the penton gene in the genome map (red box at the bottom of the image) opens a UniProt popup displaying abstracted data and a 3D structure of the encoded protein. In addition, the protein is highlighted in red in the virion image above, showing the structural role of this gene.

ViralZone pages contain many internal links to virus description pages or viral processes. A new feature has been coded throughout the resource to preview the contents of the links in a popup window when you hover over them. This has been implemented for ViralZone, Wikipedia and UniProt links (Figure 1). The popup window allows users to better understand the data without having to click on the links and load a new page.

The most popular feature of ViralZone is the rich graphical content that contains a variety of information: Virion structure, genome maps, replication cycle, molecular processes… These images were created using the vector based programme Adobe Illustrator and exported to the web format. They were in Joint Photographic Experts Group (JPG) or Portable Network Graphics (PNG) format and offered users low resolution quality and limited reusability. JPG and PNG are both raster formats and are widely supported but they have a fixed resolution and cannot support zooming without losing resolution. Scalable Vector Format (SVG), on the other hand, is a vector format that allows images to be scaled without losing detail and is supported by all browsers. We have converted more than 740 images in ViralZone to Scalable Vector Format (SVG) so that they can be scaled in size without losing quality. Moreover, users can download these images and use them in any media in the resolution (eg. theses, publications, books) without contacting ViralZone team to get a high-resolution file. All images are licenced under Creative Commons BY 4.0 (http://creativecommons.org/licenses/by/4.0); this means that they can be copied, redistributed and modified by anyone as long as the source is acknowledged.

In addition, the SVG format supports dynamic and interoperable content. Users can zoom in and out of any image, making it easier to view the internal organization of complex virions. Further interactivity is provided for virion and genome images: We coded labels in the svg files of the human viruses and added javascripts to the associated pages to add interactivity. The parts of the virion can be highlighted by mouseover, making it easier to see the global localization of each protein in the viral structure. In addition, mouseover automatically highlights the coding gene in the genome map. When hovering over a gene in the genome map, a UniProt popup appears displaying abstracted data of the encoded protein and, if available, a structure image. If this gene encodes a structural product, the corresponding proteins are highlighted in the virion image (Figure 1).

## Specific virus resources

The first specific virus resource was developed for hepatitis B virus (HBV) (9), but resources have since been developed for HIV (10), herpesviruses, coronaviruses and monkeypox virus. These resources provide detailed data focused on specific viruses of global importance. They provide further details on the molecular biology of viruses with emphasis on the following: Replication cycle, host–virus interactions, antiviral drugs, transcription and translation processes in detail.

**Figure 2.**
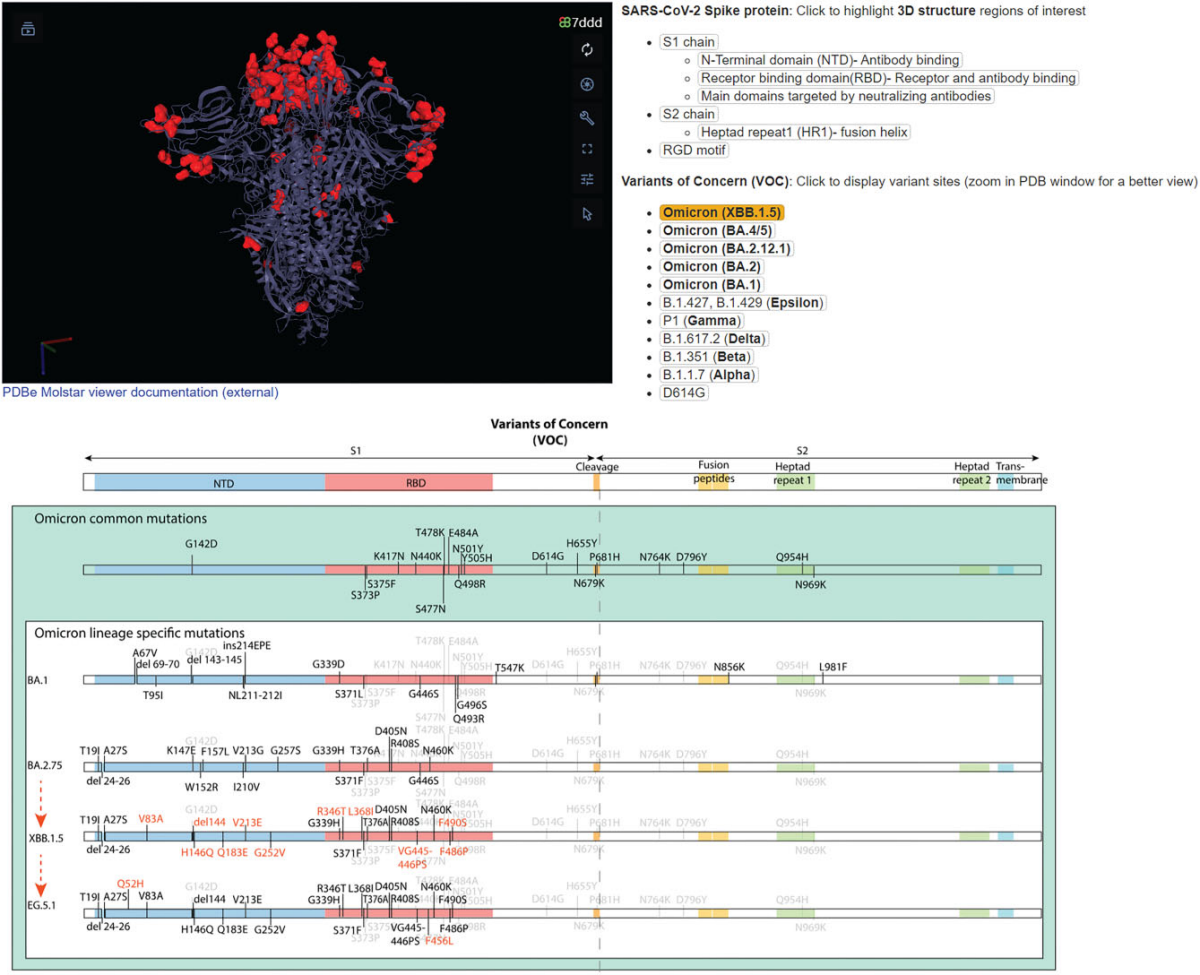
Page of SARS-CoV-2 variants. The page shows a dynamic 3D structure of the PDB:7DDD spike protein with Mol* Viewer (16). The mutations in each spike variant are highlighted in red after selecting the variant in the right list. At the bottom of the image is the graphical representation of the spike mutations present in the last major circulating variants.

The advent of COVID has highlighted the importance of rapid and accurate access to expertise on viruses. Research and medicine must have access to knowledge and data to develop accurate research, diagnostics, vaccines and therapeutics. To address this need, dedicated resources for SARS-CoV-2 viruses have been developed in ViralZone. The resource provides curated data on the biology of the virus: genome, transcriptome, proteome, enzymes and replication cycle; known antiviral drugs; vaccines; and links to epidemiological data (Nextstrain) (11). The variant page describes all major circulating variants that are or have been identified as of concern or of interest by World Health Organization (WHO) (12). The list is updated monthly as new variants of concern emerge (Figure 2). Each variant is linked several resources: CoVariants (https://covariants.org), Pango Lineages (https://cov-lineages.org), outbreak.info (13) and BV-BRC (14). Moreover, references sequences are selected for each variant in the international nucleotide sequence database (INSDC) (15) in collaboration with Nextstrain (11) and ViPR (14) so that the major virus databases provide the same references.

The Vaccines page describes the major vaccines developed during the pandemic (17), and details the vector used and the modifications added to the spike protein. The Interactome page describes the major functional interactions between hostand virus, i.e. interactions whose function has been experimentally demonstrated to play a role in the viral cycle. Many other interactions have been suggested by large-scale or preliminary experiments and can be found in external sources.

**FIGURE 3.**
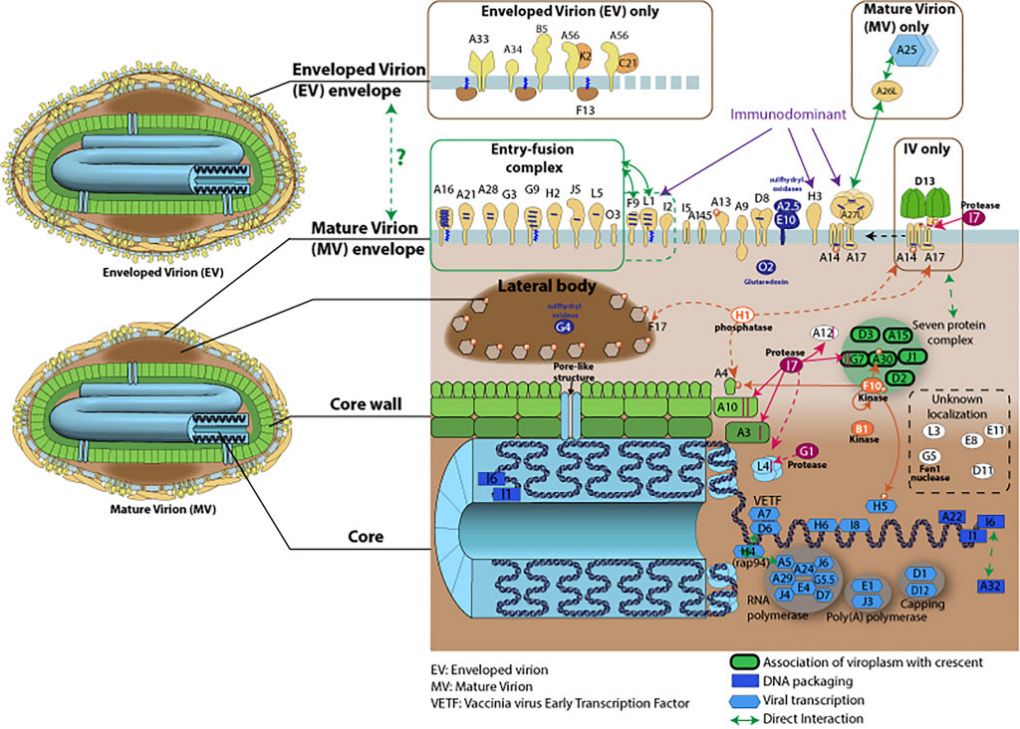
Structure of the poxvirus virion. The image shows the enveloped or mature virion with a detailed view of the internal structure with all viral proteins involved. The genes are named according to the vaccinia virus Western Reserve nomenclature.

The resources for HIV, herpesviruses, coronaviruses and monkeypox contain the same core data: Genome, proteome, host-virus interactions, replication cycle, and vaccines and antiviral drugs, as applicable. In addition, some more virus-specific information has been added: ‘HIV and Tuberculosis syndemic’ describes the mechanisms of coinfection with *Mycobacterium tuberculosis* (MTB) (18); the pages on virion organization of poxviruses and herpesviruses describe the complex internal composition of these virus particles (Figure 3) (19,20).

## Additional data update

Several updates were made throughout the database, most notably improving the data in the Human Viruses table and adding a Vertebrate Host Receptor table. The Human Viruses and associated pathologies page (https://viralzone.expasy.org/678) was updated to link all viral diseases to their corresponding Wikipedia pages, which can be previewed using the new pop-up system. In addition, genomic links have been simplified using NCBI genome assembly, which provides a single stable access to genome assembly data for monopartite or segmented genomes (21).

The Virus Host Receptor table contains comprehensive data on cellular components that function as receptors for vertebrate virus entry (https://viralzone.expasy.org/5356). Only functionally demonstrated interactions were selected, and the table contains 270 virus–receptor interactions. The table includes data on the host involved, the interacting virus-cell components, a reference publication, and additional data such as the expression of organs of the cellular receptor that can be compared with the tropism of the virus.

## Data Availability

ViralZone resource can be accessed at https://viralzone.expasy.org/. Releases are published every month. All pictures and data are licensed under a Creative Commons Attribution 4.0 International License.
